# Supplementary material for: Serum β-klotho is a potential biomarker for diagnosing alcoholic liver disease and differentiating from nonalcoholic fatty liver disease
Source: PeerJ. 2025 Aug 6;13:e19779. doi: 10.7717/peerj.19779 (PMC12335235; doi:10.7717/peerj.19779)
Supplement: Supplemental Information 4 [file peerj-13-19779-s004.docx]

AUDIT-C: Alcohol Use Disorders Identification Test consumption table (Arch Intern Med,1998,158:1789-95.)

1.How often do you drink?

口 a.never

口b.Once a month or less

口c.2~4 times per month

口 d.2-3 times a week

口e.More than 4 times a week

2.Generally speaking, how many "standard drinks" do you drink in a day? (Note: 1 standard drink = 10 grams of pure alcohol)

口 a.1 or 2 cups

口 b.3 or 4 cups

口c.5 or 6 cups

口 d.7 to 9 cups

口e.More than 10 cups

3.How often do you drink more than 6 drinks per session?

□ a.never

□ b.Less than once a month

口c.Once a month

口 d.Once a week

口e.Every day or almost every day
