# Supplementary material for: Serum β-klotho is a potential biomarker for diagnosing alcoholic liver disease and differentiating from nonalcoholic fatty liver disease
Source: PeerJ. 2025 Aug 6;13:e19779. doi: 10.7717/peerj.19779 (PMC12335235; doi:10.7717/peerj.19779)

**These data represent the result after the propensity matching.**

Supplementary Table 1 Characteristics of ALD and HC

|  | HC | ALD | *p* |
| --- | --- | --- | --- |
| n | 34 | 22 |  |
| age (mean (SD)) | 51.29 (2.79) | 48.59 (8.66) | 0.095 |
| sex = 2 (%) | 11 (32.35) | 3 (13.64) | 0.206 |
| BMI (mean (SD)) | 23.80 (2.32) | 23.74 (2.91) | 0.93 |
| sKLB (mean (SD)) | 155.28 (149.42) | 1696.21 (1119.93) | <0.001 |
| GGT (mean (SD)) | 27.94 (21.43) | 417.18 (444.17) | <0.001 |
| AST/ALT (mean (SD)) | 1.15 (0.41) | 2.40 (2.53) | 0.006 |

Supplementary Figure1 The ROC of ALD


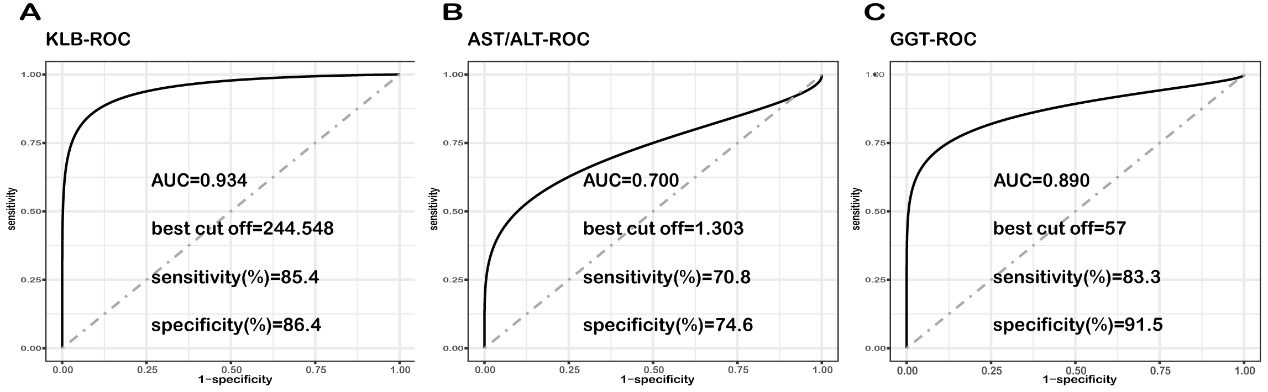


Supplementary Table2 Characteristics of NAFLD and HC

|  | **HC** | **NAFLD** | *p* |
| --- | --- | --- | --- |
| n | 43 | 30 |  |
| age (mean (SD)) | 51.49 (3.08) | 51.87 (2.75) | 0.592 |
| sex = 2 (%) | 8 (18.60) | 5 (16.67) | 1 |
| BMI (mean (SD)) | 24.64 (2.24) | 25.19 (2.73) | 0.35 |
| sKLB (mean (SD)) | 152.56 (299.08) | 75.64 (100.77) | 0.038 |

**The following is the ROC comparison of each group before propensity matching**

Supplementary Figure2 ROC curve analysis of the biomarkers in diagnosing ALD


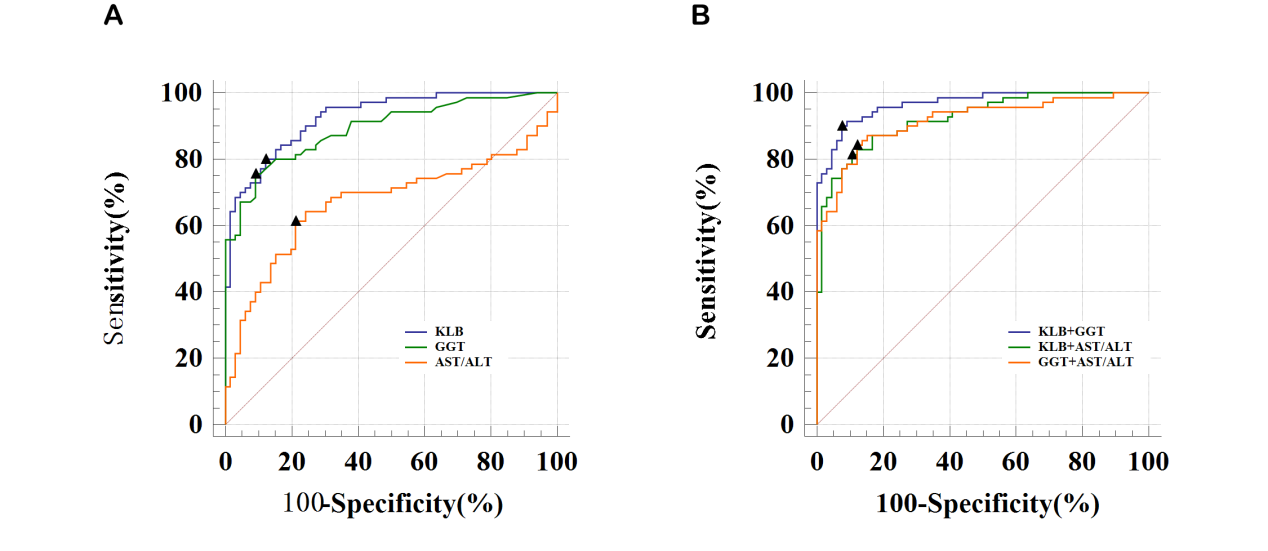


Supplementary Table3 ROC curve analysis of the biomarkers in diagnosing ALD

|  | ∆AUC | 95%CI | *p* |
| --- | --- | --- | --- |
| KLB vs GGT | 0.0351 | -0.0329-0.103 | 0.3121 |
| KLB vs AST/ALT | 0.255 | 0.154-0.356 | 0.0001 |
| KLB+AST/ALT vs GGT+AST/ALT | 0.00671 | -0.0556-0.0690 | 0.8327 |
| KLB+GGT vs GGT+AST/ALT | 0.0504 | 0.00401-0.0969 | 0.0332 |
| KLB+GGT vs KLB+AST/ALT | 0.0437 | 0.00381-0.0836 | 0.0318 |

Supplementary Figure 3 Combined diagnostic ROC curve after 2000 random draws using bootstrap internal validation


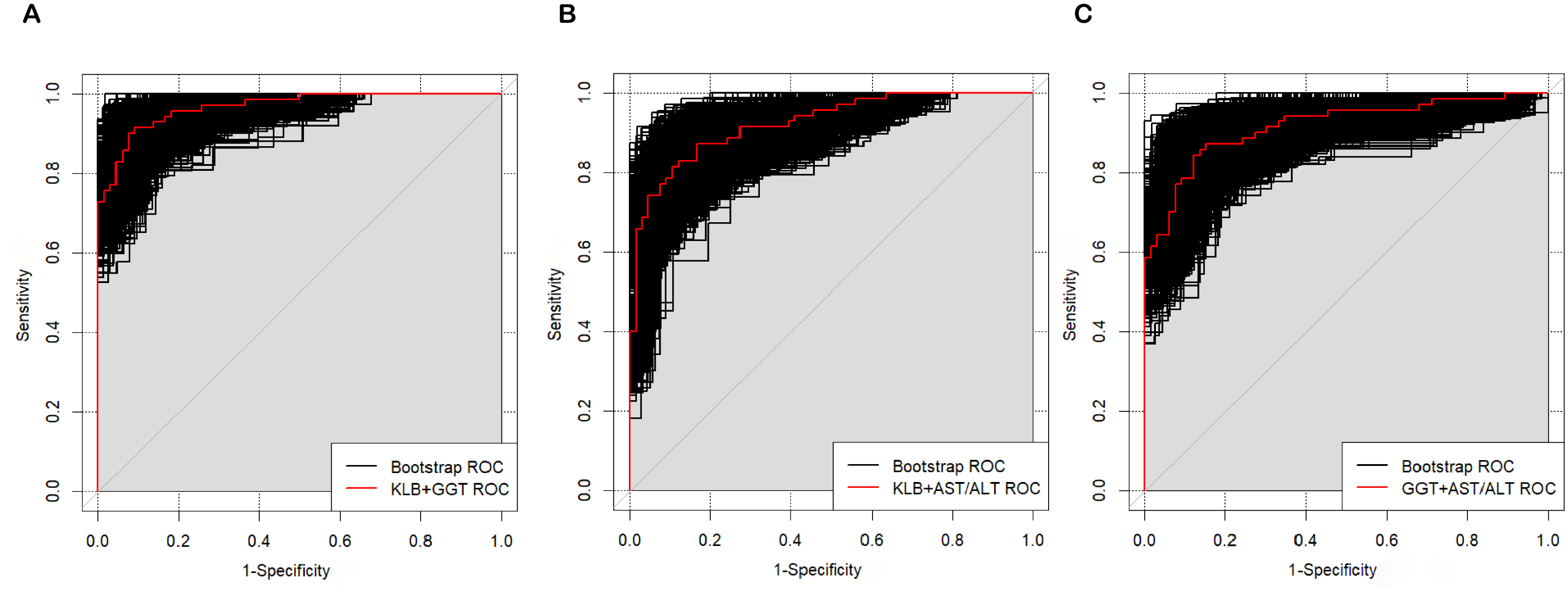

Supplement: Supplemental Information 5 [file peerj-13-19779-s005.docx]
